# Subjecting Elite Athletes to Inspiratory Breathing Load Reveals Behavioral and Neural Signatures of Optimal Performers in Extreme Environments

**DOI:** 10.1371/journal.pone.0029394

**Published:** 2012-01-19

**Authors:** Martin P. Paulus, Taru Flagan, Alan N. Simmons, Kristine Gillis, Sante Kotturi, Nathaniel Thom, Douglas C. Johnson, Karl F. Van Orden, Paul W. Davenport, Judith L. Swain

**Affiliations:** 1 Department of Psychiatry, University of California San Diego, La Jolla, California, United States of America; 2 OptiBrain Consortium, San Diego, California, United States of America; 3 Veterans Affairs San Diego Health Care System, San Diego, California, United States of America; 4 Department of Physiological Sciences, University of Florida, Gainesville, Florida, United States of America; 5 Singapore Institute for Clinical Sciences-A*STAR and National University of Singapore, Singapore, Singapore; Universidad Europea de Madrid, Spain

## Abstract

**Background:**

It is unclear whether and how elite athletes process physiological or psychological challenges differently than healthy comparison subjects. In general, individuals optimize exercise level as it relates to differences between expected and experienced exertion, which can be conceptualized as a body prediction error. The process of computing a body prediction error involves the insular cortex, which is important for interoception, i.e. the sense of the physiological condition of the body. Thus, optimal performance may be related to efficient minimization of the body prediction error. We examined the hypothesis that elite athletes, compared to control subjects, show attenuated insular cortex activation during an aversive interoceptive challenge.

**Methodology/Principal Findings:**

Elite adventure racers (n = 10) and healthy volunteers (n = 11) performed a continuous performance task with varying degrees of a non-hypercapnic breathing load while undergoing functional magnetic resonance imaging. The results indicate that (1) non-hypercapnic inspiratory breathing load is an aversive experience associated with a profound activation of a distributed set of brain areas including bilateral insula, dorsolateral prefrontal cortex and anterior cingulated; (2) adventure racers relative to comparison subjects show greater accuracy on the continuous performance task during the aversive interoceptive condition; and (3) adventure racers show an attenuated right insula cortex response during and following the aversive interoceptive condition of non-hypercapnic inspiratory breathing load.

**Conclusions/Significance:**

These findings support the hypothesis that elite athletes during an aversive interoceptive condition show better performance and an attenuated insular cortex activation during the aversive experience. Interestingly, differential modulation of the right insular cortex has been found previously in elite military personnel and appears to be emerging as an important brain system for optimal performance in extreme environments.

## Introduction

The neuroscience underlying optimal performance in extreme environments is in its infancy [1]. Nevertheless, there is a burgeoning interest in understanding how the brain contributes to optimizing performance [2]. Altered cortical and subcortical processing of tasks and external conditions has been proposed as an important mechanism that differentiates elite performers from comparison subjects [3]. In a prior study, we examined neural processing of elite military personnel (U.S. NAVY Sea, Air, and Land Forces–SEALs) relative to comparison subjects during emotion face processing, and showed relatively greater right-sided insula, but attenuated left-sided insula, activation in the elite performers. Moreover, the U.S. Navy SEALs showed selectively greater activation to angry target faces relative to fearful or happy target faces in both right and left insula [4]. These individuals also show greater insula activation when anticipating a change in interoceptive state from the current state, but reduced insula activation to aversive images relative to comparison subjects (Simmons, in prep). Taken together, these results are consistent with the hypothesis that elite performers deploy processing resources that are more focused on specific task demands, and they are better able to respond to external stimuli that perturb internal homeostasis.

Interoception comprises the sensing of the physiological condition of the body [5], the representation of this internal state [6] within the context of ongoing activities, and the initiation of motivated action to homeostatically regulate the internal state [7]. Interoception is an important process for optimal performance because it links the perturbation of internal state as a result of external demands to goal-directed action that maintain a homeostatic balance [8]. In particular, the interoceptive system provides information about the internal state to neural systems that monitor value and salience and are critical for cognitive control processes. We recently proposed that maintaining an interoceptive balance by generating body prediction errors in the presence of significant perturbations may be a neural marker of optimal performance [8]. This notion is consistent with findings that elite athletes pay close attention to bodily signals [9] and may be particularly adept in generating anticipatory prediction errors [10]. Further, others [11] have proposed that individuals regulate performance via perceived exertion through a “teleoanticipation” process [12] which is the combination of afferent and efferent brain processes that attempt to couple the metabolic and biomechanical limits of the body to the demands of the exercise task. Specifically, an individual's expectation of effort perception during exercise is the basis for an ongoing interpretation of perceived exertion, and is due to both efferent feed-forward and afferent feedback signals [12]. Thus, neural systems that process the internal body state and are able to generate small body prediction errors may be critical for optimal performance.

An extreme environment can be defined as an external context that exposes individuals to demanding psychological and/or physical conditions, and which may have profound effects on cognitive and behavioral performance [8]. Examples of these types of environments include combat situations, Olympic-level competition, and expeditions in extreme cold, at high altitudes, or in space. Adventure racing is a combination of two or more endurance disciplines such as orienteering, navigation, cross-country running, mountain biking, paddling, climbing, and related rope skills. Individuals participating in adventure racing experience significant physical and psychological stress during these competitions, which sometimes result in both significant injury and in mood-state disruption [13]. In this study we examine elite adventure racers who are non-military elite performers and who are often exposed to extreme environments [14].

The sensation of breathing is a complex process that is modulated by numerous factors [15]. In addition to chemoreceptors that form reflex feedback mechanisms for respiratory motor activities [16], breathing is also influenced by internal and external environmental changes, which is termed behavioral breathing. Respiratory sensations are an essential interoceptive experience because there is a profound evaluative component associated with breathing sensation, and there is a strong motivational aspect to adjust breathing via the respiratory motivation-to-action neural system [17]. These sensations are the result of both subcortical and cortical processes [18], which include discriminative processing (the awareness of the spatial, temporal and intensity components of the respiratory input) and affective processing (the evaluative and emotional components of the respiratory input).

Resistive load, i.e. restricted inspiration, was first introduced by Lopata [19] and Gottfried [20], and is an airflow-dependent load [21] and a simple but powerful experimental approach to induce an altered interoceptive state. In contrast to expiratory breathing load which affects C0_2_ [19], inspiratory breathing load results in stable, unchanged C0_2_ levels [22]. Inspiratory breathing loads can be used to examine breathing difficulty and generate respiratory-related evoked potentials with several peaks that indicate the transition from an early sensory component to a later cognitive aspect [23], [24], [25], [26]. Moreover, resistive loads generate pre-motor potentials that reflect the involvement of higher cortical motor areas [27], they decrease systolic blood pressure [28], they differ for males and females [29], they are perceived less intense in older individuals [30], they generate load-dependent increases of unpleasantness [31], and the subjective effects can be modified by attentional distractions [32]. Thus, inspiratory breathing load provides a powerful experimental approach to examine how optimal performers respond to the temporary perturbation of the internal body state.

We have previously proposed that optimal performance may be related to the ability to effectively minimize the body prediction error [8], which allows individuals to better adjust to environmental perturbations. Since the insula cortex is important in generating body prediction errors [33], then one would hypothesize that elite athletes show attenuated neural processing in the insular cortex of afferent aversive interoceptive stimuli. Support for this hypothesis would provide further evidence that elite performers show a distinct brain signature that enables them to adjust more quickly and appropriately to extreme environments. This approach uses simple laboratory tasks to link neural and cognitive processes that have been found to be important for elite performance. As pointed out by others, this approach may help to explain sporting skill at the highest levels of performance [1].

## Methods

### Participants

The University of California San Diego (UCSD) Institutional Review Board approved this study and all subjects signed informed consent. Ten adventure racers (6 males, 4 females) were recruited by word of mouth and fulfilled the following criteria: (1) participated in multi-day events on an international level; (2) placed among the top 5 performing teams in at least 3 races; (3) completed international races within the past 5 years; (4) were at least 14 days out from their last race. The last criterion was used to minimize acute effects related to physical and psychological exhaustion. Eleven healthy control subjects (8 males, 3 females) were recruited from other ongoing studies supported by the Center of Excellence for Stress and Mental Health (CESAMH). All twenty-one subjects completed the study. The mean age of the adventure racers was 37.5+/−6.0 years, and the controls 36.6+/−6.9 years. The adventure racers completed 16.3+/−1.8 years of education, and the control subjects 16.0+/−1.4 years of education. The groups did not differ in gender (χ^2^ = 0.382, *p* = 0.537), age (*t*(19) = 0.30, *p* = 0.76), or years of education (*t*(19) = 0.29, *p* = 0.77). All subjects were trained to perform a non-hypercapnic breathing load task prior to fMRI scanning. No restrictions were placed on the consumption of caffeinated beverages prior to study, and none of the subjects were smokers.

### Measures

Several personality and symptom assessment questionnaires were administered including the Sensation Seeking Scale [34], the Barratt Impulsiveness Scale [BIS-11, 35], the Brief Symptom Inventory (BSI), a brief psychological self-report symptom scale [36], and the Connor Davidson Resiliency Scale CD-RISC [37].

### Aversive Interoceptive Stimulus: Non-Hypercapnic Inspiratory Breathing Load

The subjects wore a nose clip and breathed through a mouthpiece with a non-rebreathing valve (2600 series, Hans Rudolph) that maintains an airtight seal. The apparatus was attached to the scanner head coil to eliminate the need for the subject to contract mouth muscles. The resistance loads consisted of sintered bronze disks placed in series in a Plexiglas tube (loading manifold), with stoppered ports inserted between disks. Loads were selected by removing the stopper and allowing the subject to inspire through the selected port. Each subject was given the following instructions: “This task examines how people feel when breathing becomes difficult. You will breathe through a hose, which makes breathing-in more difficult. It is important for you to know that this test is not physically harmful, but you may feel uncomfortable when you breathe through the hose. You can stop at any time if breathing becomes too difficult. You will be asked to breathe through the hose several times. We would like you to complete a one-page rating scale after each trial”. Based on preliminary data and previous experience, 40 cmH_2_O/L/sec was selected as load, which alters subjective symptoms without significantly affecting CO_2_ or O_2_ level. The subjects were asked to rate their experience on a 10 cm Visual Analog Scale which was anchored from “not at all” to “extremely” on the following 16 dimensions: pleasant; unpleasant; intense; tingling; fear of losing control; faintness; fear of dying; unreality; hot/cold flushes; trembling; choking; abdominal distress; chest pain; palpitations; sweating; and dizziness, all of which correspond to items used in one author's (PWD) prior studies [17], [38].

The basic experimental approach was similar to that of a recently published study by our group involving human touch [39]. Specifically, individuals performed a simple continuous performance task during the paradigm. Subjects were asked to press a button corresponding to the direction pointed by an arrow on the screen (left arrow = left button, right arrow = right button). Both accuracy and response latency were recorded and analyzed to determine effects of anticipation and stimulus presentation. At the same time, the background color of the stimulus served as a cue to the impending presentation of the breathing load, with gray indicating that there will be no load, and yellow indicating a 25% chance of load. Throughout the task, subjects experienced 3 conditions: (1) baseline condition: the individual performs the continuous performance task; (2) anticipation condition: the background color behind the arrow signals an impending restricted breathing period; and (3) stimulus condition: during the change in background color there is a 25% probability that the subject experienced a 40 second period of restricted breathing. We introduced this probability to maximize the opportunity to measure the effect of anticipating an aversive interoceptive event. The implementation of this paradigm utilized an event-related fMRI design consisting of 2 scans with 256 repetitions (TR = 2 secs) yielding a total scan duration of 17 minutes and 4 seconds. During this period the individual was presented with 32 anticipation conditions during which 8 breathing-load episodes occurred. The duration of each condition is “jittered” in time to permit optimal resolution of the hemodynamic response function. On average the baseline condition lasted 9 seconds, the anticipation condition 9 seconds, and post-stimulus condition 12 seconds. Throughout the baseline condition a black arrow on a gray background was presented on the screen every 3 seconds. For the anticipation condition, subjects were informed that a blue background on the screen predicted the subsequent restricted breathing period; this phase lasted between 6 and 12 seconds. The main behavioral variable was performance accuracy and latency during the three different stimulus conditions, and the main neuroimaging-dependent measure was the activation in functionally constrained regions of interest during the anticipation and stimulus condition relative to the baseline condition.

### Neuroimaging analyses

#### Acquisition of images

Imaging experiments were performed on a 3T GE CXK4 Magnet at the UCSD Keck Imaging Center, which is equipped with 8 high bandwidth receivers that allow for shorter readout times and reduced signal distortions and ventromedial signal dropout. Each one hour session consisted of a three-plane scout scan (10 seconds), and a standard anatomical protocol consisting of a sagittally acquired spoiled gradient recalled (SPGR) sequence (FOV 25 cm; matrix: 192×256; 172 sagittally acquired slices thickness: 1 mm; TR: 8 ms; TE: 3 ms; flip angle = 12). We used an 8-channel brain array coil to axially acquire T2*-weighted echo-planar images (EPI). The parameters for the EPI scans were: FOV 230 mm, 64×64 matrix; 40 2.6 mm thick slices; 1.4 mm gap; TR = 2000 ms, TE = 32 ms, flip angle = 90°. Rapid image acquisition was obtained via GE's ASSET scanning, a form of sensitivity encoding (SENSE) which uses parallel imaging reconstruction to allow for sub k-space sampling.

#### Image analysis pathway

All subject-level structural and functional image processing was done with the Analysis of Functional Neuroimages (AFNI) software package [40]. The multivariate regressor approach detailed below was used to relate changes in EPI intensity to differences in task characteristics [41]. EPI images were co-registered using a 3D-coregistration algorithm [42] that was developed to minimize the amount of image translation and rotation relative to all other images. Six motion parameters were obtained across the time series for each subject. Motion parameters were used as regressors to adjust EPI intensity changes due to motion artifacts. This has been shown to increase power in detecting task-related activation [43]. All slices of the EPI scans were temporally aligned following registration to assure that different relationships with the regressors are not due to the acquisition of different slices at different times during the repetition interval.

#### Multiple regressor analyses

Regressors of interest were generated to delineate the three conditions described above: (1) anticipation, (2) breathing load, and (3) a post-breathing interval. To that end, a 0–1 reference function of the particular time interval was convolved with a gamma variate function [44] modeling a prototypical hemodynamic response (6–8 second delay [45]) and to account for the temporal dynamics of the hemodynamic response (typically 12–16 seconds) [46]. The convolved time series was normalized and used as a regressor of interest. A series of regressors of interest and the motion regressors were entered into the AFNI program 3dDeconvolve to determine the height of each regressor for each subject. The main dependent measure was the voxel-wise normalized relative signal change, or % signal change for short. Subsequently spatial smoothing with 6 mm FWHM was applied to the % signal change data, which were transformed into Talairach coordinates based on the anatomical MR image for group or second-level analysis.

#### Group level analyses

For the interoceptive fMRI paradigm the dependent measure was the % signal changes during the anticipation, stimulation, and post-stimulation periods, respectively. These dependent measures were entered into a *mixed effects model* [47]. We used the implementation of the linear mixed effects models in R (www.cran.org), which estimates the parameters of the mixed model using Maximum Likelihood Estimation (MLE). These calculations were done within the R computing environment using routines that read in AFNI data sets. Specifically, the group (elite athletes versus comparison subjects), and the experimental conditions (anticipation, stimulation, post-stimulus interval) were used as a fixed factor, and subject was used as a random factor. The effects are estimated using specific contrast matrices. Once these voxel-wise statistics were calculated, we used a threshold adjustment method based on Monte-Carlo simulations to guard against identifying false positive areas of activation. Based on simulations implemented in the AFNI program AlphaSim, by using a constrained region of interest analysis approach for the insular cortex it was determined that the volume threshold for clusterwise probability of 0.05 was 512 uL. Only these clusters were considered for further analysis. Finally, we conducted voxel-wise multiple linear regression analyses with self-report measures as independent measures, and the percent signal change during the breathing load condition as the dependent measure using the robust Huber regressions based on the rlm program of R.

## Results

### Behavioral results

Adventure racers relative to comparison subjects showed elevated self-ratings of sensation seeking (Table 1). With the exception of the thrill and adventure seeking subscale of the Sensation Seeking Scale, adventure racers rated higher on all other subscales. There were no significant overall differences between adventure racers and controls on the Barratt Impulsivity Scale, however, adventure racers rated themselves higher on the perseverance subscale. Finally, there were no differences between adventure racers and comparison subjects on the Brief Symptoms Inventory or on the Connor Davidson Resiliency Scale.

**Table 1:** Personality and symptom assessment of elite athletes and comparison subjects.

	Athlete		Control	
	mean	std	mean	std
**Sensation Seeking Scale**	27.77**	4.23	20.58	3.98
Thrill and Adventure Seeking (TAS)	9.33	1.11	7.92	2.39
Experience Seeking (ES)	7.44*	1.66	5.91	1.62
Disinhibition (Dis)	7**	1.93	4.41	2.35
Boredom Susceptibility (BS)	4**	1.65	2.33	1.23
**BIS-11**	74.64	6.16	70.75	5.62
Attention	12.75	2.54	11	2.18
Motor	16.5	3.7	15.08	1.75
Self-Control	15.87	3.48	16.67	2.42
Cognitive Complexity	12.75	2.37	12.5	2.49
Perseverance	9.62**	1.3	7.58	1.48
Cognitive Instability	7.125	2.41	6	1.56
**BSI-18**	2.77	3.23	2.42	2.17
**CDRISC**	30.66	12.32	31.42	10.07
	** p<0.01			
	* p≤0.05			

Personality and symptom assessments show that elite athletes score higher on sensation seeking and perseverance than comparison subjects.

### Self-report during breathing load

**Figure 1:**
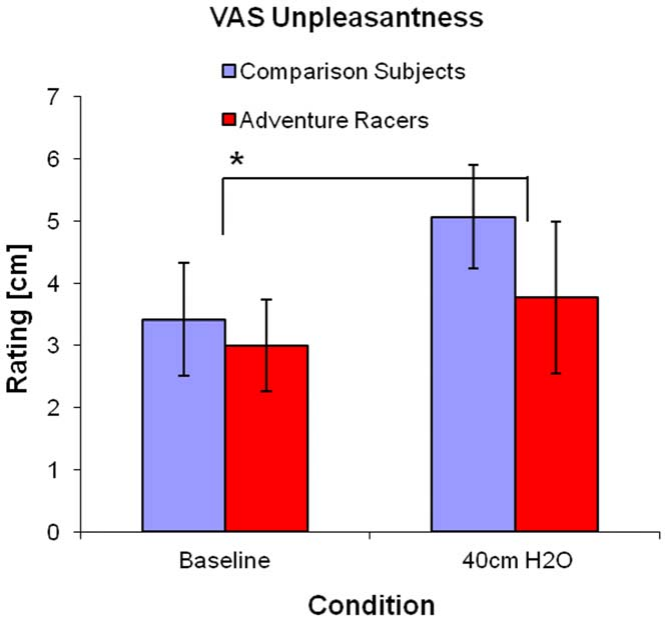
Visual Analog Rating during Baseline (no load) and 40 cm H2O/L/sec inspiratory breathing load in comparison subjects and elite athletes, respectively. Both groups showed increased unpleasantness during the 40 cm H2O/L/sec load condition.

There was an overall increase in VAS scale ratings of unpleasantness when comparing baseline to 40 cm H2O/L/sec load [F(1,15) = 7.427, p = 0.0156] (Figure 1). However there were no significant group differences [F(1,17) = 0.642, p = 0.4339] or group by condition interaction [F(1,15) = 0.126, p = 0.7273]. Although the breathing load resulted in an aversive experience, there were no differences in the degree of unpleasantness between adventure racers and comparison subjects.

### Behavioral performance during breathing load

**Figure 2:**
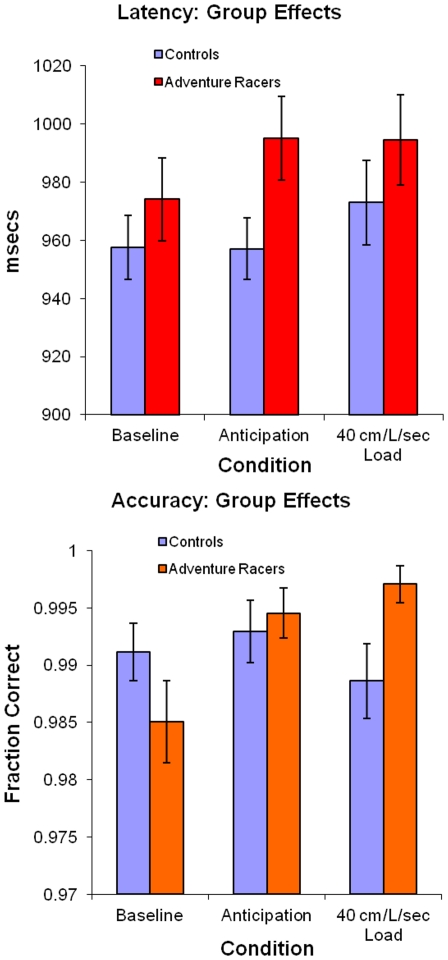
Behavioral performance (latency and accuracy) during the continuous performance task in both comparison subjects and athletes (left) and separately for each group (right).

Individuals took longer to select a response during the anticipation and during the breathing load conditions [F(2,95) = 6.242, p = 0.0028](Figure 2). Adventure racers did not differ from comparison subjects [F(1,19) = 1.034 p = 0.322] and there was no differential effect of anticipation or load condition for the elite athletes relative to the controls [F(2,95) = 2.441 p = 0.0925]. In contrast, there was an overall trend for increased response accuracy between groups during the anticipation and breathing load conditions [F(2,95) = 2.8 p = 0.0630]. More specifically, whereas healthy volunteers showed no clear difference in accuracy during the different conditions, adventure racers showed greater response accuracy during the anticipation and breathing load condition, resulting in a significant group-by-condition interaction [F(2,95) = 4.5, p = 0.0136]. Thus, the aversive interoceptive perturbation improved performance in adventure racers but not in healthy controls.

### Neuroimaging results

#### Task Effect

**Figure 3:**
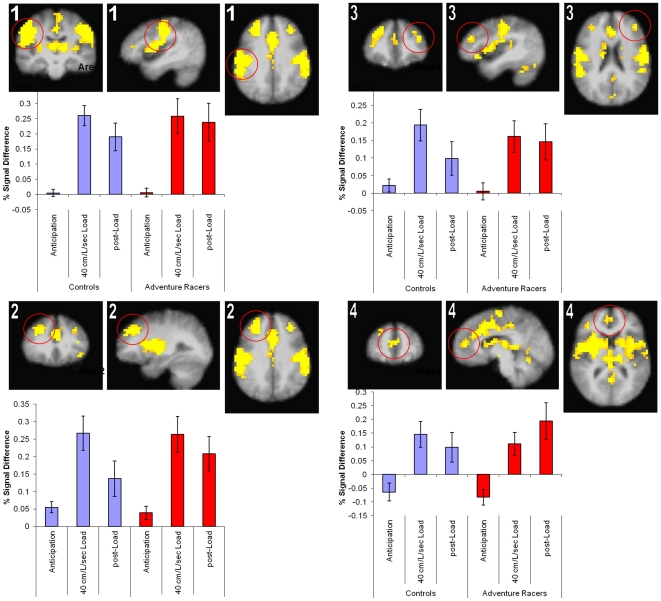
Main effect of task, i.e. brain changes as a consequence of inspiratory breathing load in both comparison subjects and elite athletes. Activation increases primarily during the breathing load and post-breathing load condition.

Loaded breathing induced a large change in brain activation that varied across task condition (Table 2), and which affected several areas of the brain as shown in Figure 3. In general breathing load resulted in significant activation increases in the bilateral insular cortex, anterior cingulate, and also in the bilateral dorsolateral prefrontal cortex. In each of these areas activation was significantly greater during the breathing load and post-breathing load period relative to the anticipation period. Moreover, both adventure racers and healthy comparison subjects showed similarly strong activation across the different experimental conditions.

**Table 2:** Main effect of breathing restriction on brain activation in comparison subjects and elite athletes.

Volume	x	y	z	Brain Area	BA
154624	2	−13	22	Bilateral Cingulate Gyrus	BA 23
7424	29	36	33	Right Superior Frontal Gyrus	BA 9
2304	−36	37	29	Left Middle Frontal Gyrus	BA 9
1984	3	49	14	Right Medial Frontal Gyrus	BA 10
1408	16	−89	−17	Right Declive	BA 18

Volume (µL), center of mass coordinate, and brain area based on the voxel-wise mixed model main effect of breathing load. These areas showed brain activation related to loaded breathing for both comparison subjects and elite athletes.

#### Task×Group Interactions

**Figure 4:**
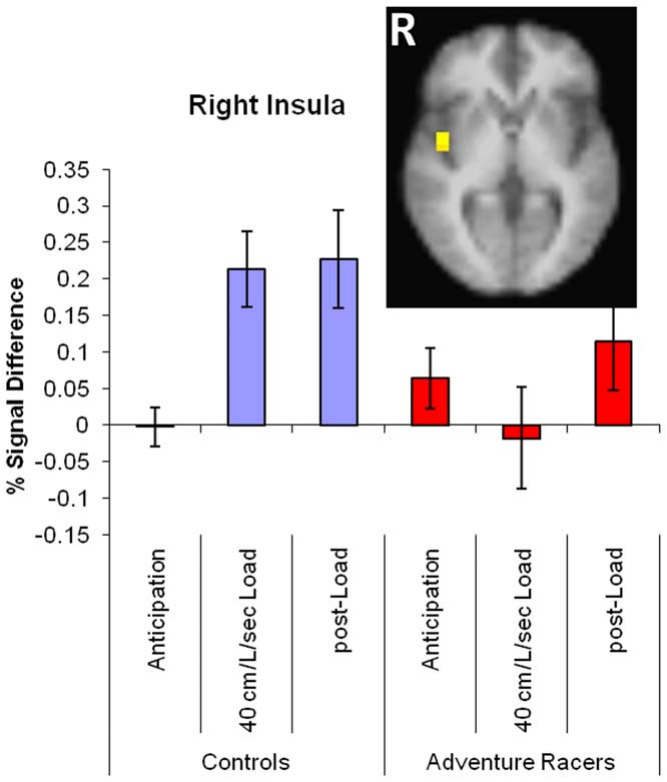
Group×Task Interaction, right middle insula showed significantly greater activation during breathing load and post-breathing load condition in comparison subjects relative to elite athletes.

Using the constrained region of interest analysis, the right insular cortex (Figure 4 and Table 3) was the only brain area that showed a significant task-by-group interaction, with adventure racers relative to comparison subjects differentially activating this brain area as a function of task condition. The right insular cortex was found to activate during the task in general [F(1,38) = 11.295, p = 0.0018]. Specifically, relative to the anticipation condition, both groups showed greater activation during the breathing load and post-breathing load conditions [F(2,38) = 5.890, p = 0.0059]. Moreover, there were no overall group differences between adventure racers and comparison subjects (F(1,19) = 2.977, p = 0.1007). Importantly, whereas healthy volunteers showed an overall increase in activation during the load condition, adventure racers showed greater activation during the anticipation phase and attenuated activation during the load phase, resulting in a significant condition-by-group interaction [F(2,38) = 6.184 p = 0.0047]. Finally, activation of the right insular cortex during the breathing load condition correlated negatively with performance accuracy (r = −048, p = 0.02), with greater activation resulting in lower performance accuracy. There was no significant correlation of right insular cortex activation with latency or with the subjective rating of unpleasantness (all ps>0.05).

**Table 3:** Task by group interaction: Elite athletes relative to comparison subjects differentially activated the right insula cortex.

Volume (uL)	x	y	z	Area	BA
1152	44	−2	1	Right Insula	BA 13

Volume (µL), center of mass coordinate, and brain area based on the voxel-wise mixed model task by group interaction. There were significant differences in the right insula cortex between adventure racers and comparison subjects.

#### Brain behavior relationships

**Figure 5:**
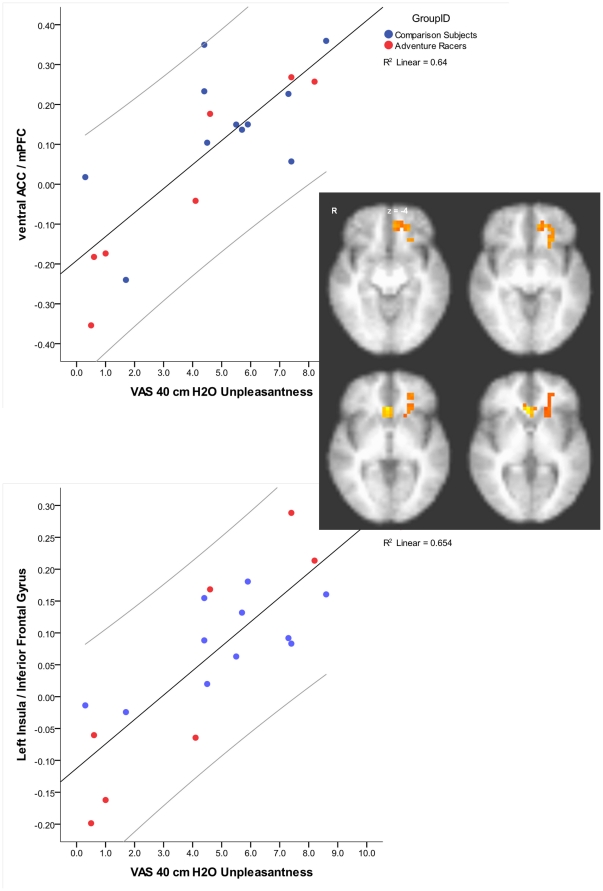
Brain activation during breathing load and self-rating of unpleasantness correlations in both comparison subjects and elite athletes. Those individuals who rated the load as more unpleasant also showed greater activation in ventral ACC and left insula.

Whole brain analyses using robust regression with both groups revealed that the degree of brain activation during breathing load in two brain areas correlated with the subjective ratings of unpleasantness due to breathing load. Specifically, ventral anterior cingulate and left anterior insula (including lateral inferior frontal gyrus) showed greater activation in those subjects with higher unpleasantness ratings (Figure 5). There were no differences across groups in these areas. Further analysis of the brain area that correlated with self-rated unpleasantness revealed two additional relationships. First, higher impulsiveness ratings were associated with lower activation in the left anterior insula during breathing load (r = −0.46, p = 0.04). Second, greater activation during anticipation in the right anterior insula was associated with less activation in the left anterior insula during breathing load (r = −0.53, p = 0.01). There were no correlations between either the ventral anterior cingulate area or the insula area and measures of performance (accuracy or latency) during the continuous performance task.

## Discussion

We examined the hypothesis that elite athletes show attenuated neural processing of aversive interoceptive stimulation in the insular cortex by testing the behavioral and neural processing response of elite athletes during an aversive interoceptive non-hypercapnic breathing load. The experiment yielded three main results. First, non-hypercapnic inspiratory breathing load is an aversive experience that results in a profound activation of a distributed set of brain areas including bilateral insula, dorsolateral prefrontal cortex and anterior cingulate. The degree of activation in a subset of brain areas consisting of the ventral anterior cingulate and left anterior insula was correlated with subjective ratings of unpleasantness. Second, adventure racers, compared with control subjects, show greater accuracy on the continuous performance task during the aversive interoceptive stimulation. Third, adventure racers show attenuated right insula cortex response during the breathing load and the post-breathing condition. Taken together, this experimental approach not only shows that insular activation differentiates elite athletes (as an example of optimal performers) from comparison subjects, but also shows that these individuals perform better during aversive interoceptive stimulation on a simple continuous performance task. Thus, non-hypercapnic breathing load during functional neuroimaging provides a laboratory approach to study elite performers and identify behavioral and brain processes that are important for optimal performance in extreme environments.

Adventure racing at an elite level comprises competitions that last 100 hours or longer and can cause physical injury and perturbation of mood states, which in turn can have profound impact on optimal performance [13]. Measures of mood states have been used to predict athletic injury [48], but much less is known about the central nervous system contribution to optimal performance. There is a growing interest in understanding how basic brain processes influence levels of performance, and several investigators have begun to delineate which brain processes contribute to athletic performance [49], [50]. The finding in this study identifies a brain area (the right insula) and a process (the response to aversive interoceptive stimulation) that differ in elite athletes. The finding is consistent with the proposed role of the insular cortex as a component of the so-called central command, i.e. the brain systems that are important for modulating the degree to which individuals engage in demanding athletic performance [49], [51]. Several neuroimaging studies using Single Photon Emission Tomography during physical exercise have demonstrated changes in activation in the insula cortex. For example increased left insula regional cerebral blood flow (rCBF) was observed during active, but not passive, cycling [52]. Moreover, greater insular rCBF was positively correlated with levels of perceived cycling intensity [53] and with individual blood pressure changes. Therefore, there is an emerging role of the insular cortex in processing effort as the athlete perceives it during exercise and modulating physiological parameters that are critical for optimizing physical performance.

The relationship between self-rated unpleasantness and the degree of activation in ventral anterior cingulate, left anterior insular and lateral inferior frontal gyrus supports the notion that brain structures which are important for regulating subjective mood states [54], [55] are also critical for modulating optimal performance. These findings are consistent with those of Williamson and colleagues who have used false feedback of less than or greater than actual physical demand to examine central nervous system regulation of perceived exertions. These investigators found that under these conditions, changes in rCBF in left and right insular cortex as well as anterior cingulate cortex correlated with perceived exertion, but not with changes in heart rate or blood pressure [56]. Furthermore, both insular and anterior cingulate cortices were also found to activate during imagined exercise [57]. Taken together, the insula and anterior cingulate cortex are important for processing levels of exertion with false feedback and in an imaginary condition, i.e. can function as a central command system without the necessity of peripheral feedback. The insula and anterior cingulate also interact with thalamic and brainstem structures which are important for cardiovascular integration. Therefore, both insula and anterior cingulate may process the individual's sense of effort or exertion with and without the need for peripheral afferents [49].

The differential activation in the right insular cortex in elite adventure racers during breathing load and in the post-breathing condition is similar to the differential activation during an emotion processing task in NAVY SEALs relative to male comparison subjects that we previously reported [4]. Although there are a number of caveats, e.g. there were different task and conditions, different selective demand-dependent activations, and different genders of subjects, there are some common findings between these two studies that deserve to be highlighted. In both conditions elite performers showed relatively less activation in conditions that were “more challenging” to healthy volunteers. In extension of the previous study that did not show performance accuracy or response latency differences between elite war fighters and comparison subjects [4], the current study shows that elite athletes demonstrate greater accuracy under challenging conditions. The combination of a continuous performance task and non-hypercapnic inspiratory breathing load may provide a simple behavioral probe to examine both brain processing and behavioral performance differences in individuals who are training to acquire elite performance status. Moreover, the brain-behavior relationship between the insular cortex and self-rated unpleasantness and task performance may provide an initial step toward development of a peripheral biomarker of optimal performance.

We have recently proposed a neuroanatomical processing model as a heuristic guide to understand how interoceptive processing may contribute to optimal performance. In this model we propose that optimal performers generate more efficient body prediction errors, i.e. the difference between the value of the anticipated/predicted and value of the current interoceptive state, as a way of adapting to extreme environments. However, body prediction error differences may occur on several levels. For example, optimal performers may receive different afferent information via the C-fiber pathway that conveys spatially- and time-integrated affective information [7]. Alternatively, optimal performers may generate centrally different interoceptive states (e.g., via contextual associations from memory), which are processed in insular cortex via connections to temporal and parietal cortex to generate body states based on conditioned associations [58]. Consistent with this idea, Williamson and colleagues suggest that the neural circuitry underlying central regulation of performance includes the insular and anterior cingulate cortex that interact with thalamic and brainstem structures which are important for cardiovascular integration [49] as well as for the central modulation of cardiovascular responses [57].

Optimal performers may also differentially integrate interoceptive states within the insular cortex (which shows a clear gradient from the dorsal-posterior to ventral-anterior part) to provide an increasingly “contextualized” representation of the interoceptive state [59]. This integration may occur irrespective of whether it is generated internally or via the periphery. The relative increase in activation in the mid-insula in adventure racers prior to experiencing the breathing load, and the relatively attenuated activation after the load experience, support the notion that the aversive interoceptive experience is less disruptive to these elite athletes compared to control subjects, and may lead to relatively fewer changes in the subjective response to this stressor.

Optimal performers may generate different context-dependent valuation of the interoceptive states within the orbitofrontal cortex [60] leading to altered error processing in the anterior cingulate [61] and selection of different actions [62]. The findings that both ventral anterior cingulate and left anterior insula response are important for the subjective effects of the breathing load support the notion that optimal performers may show different integration of aversive interoceptive stimuli. These results are consistent with those of Hilty and colleagues [63] who reported that individuals who perform a handgrip exercise prior to task failure show increased activation in both the mid/anterior insular cortex and the thalamus. Thus, greater activation and possibly a larger body prediction error might predict sub-optimal performance.

Finally, it is also unclear whether optimal performers generate different learning signals (similar to reward prediction error [64]), as part of the interactions between the insula and the basolateral amygdala [65] and the ventral striatum [66]. The current results are consistent with the notion that integration within different parts of the insula cortex as well as top-down, feed-forward information from other brain areas are important to optimize performance. Optimal performers are able to more quickly adapt to both bottom-up interoceptive afferents and top-down cognitive control brain areas that modulate mood and anxiety [67] in regulating one's response to an aversive interoceptive perturbation. Future investigation will need to examine at what stage of the pathway elite athletes or optimal performers differ from comparison subjects. This will require not only studies with more subjects but also different paradigmatic approaches. Nevertheless, by disentangling the processes that contribute to optimal performance one can begin to develop brain-process specific interventions that aim to improve performance.

The central governor model focused on perceived exertion [68] (the subjective perception of exercise intensity) has been used to explain performance differences in athletes [69]. Recently this model has been extended by Tucker and colleagues [11] based on prior formulations by Hampson [12]. Specifically, a system of simultaneous efferent feed-forward and afferent feedback signals are thought to optimize performance by overcoming fatigue through permitting continuous compensation for unexpected peripheral events [12]. Afferent information from various physiological systems and external or environmental cues at the onset of exercise can be used to forecast the duration of exercise within homeostatic regulatory limits. This enables individuals to terminate the exercise when the maximal tolerable perceived exertion is attained. In this model the brain creates a dynamic representation of an expected exertion against which the experienced exertion can be continuously compared [11] to prevent exertion from exceeding acceptable levels. The notion of a differential between expected and experienced exertion parallels our model of the body prediction error [8]. However, the degree to which peripheral input is necessary is still under debate. For example, Marcora and colleagues have developed a psychobiological model which proposes that perceived exertion is generated by a top-down or feed-forward signal [50], i.e. the brain – not the body – generates the sense of exertion. These investigators have argued that the a centrally generated corollary discharge of the brain is critical for optimal effort [70], and that mental fatigue affects performance via altered perception of effort rather than afferent and body originating cardiorespiratory and musculoenergetic mechanisms [71]. Nevertheless, whether it is a purely central process, as suggested by Marcora, or an interaction between afferent peripheral feedback and efferent central feed-forward systems, the differential between the expected and observed, i.e. the body prediction error, is the critical variable that moderates performance. The implementation of this process in the brain and its modulation by nature or nurture will be central to understand optimal performance.

This investigation had several limitations. First, the group of elite athletes we studied was relatively small and thus there may be a lack of power to detect additional behavioral/functional relationships. With larger number of subjects and different tasks, other important relationships may become apparent. Second, this cross-sectional study could not address the question of whether the observed processing differences were part of the preexisting characteristics of individuals who were selected and then trained to become elite athletes, or whether these neural processing differences were a consequence of training. Thus, future studies will need to examine, in a within-subjects study design, individuals prior to and again after elite athlete training.

This systems neuroscience approach to understanding optimal performance in extreme environments has several advantages over traditional descriptive approaches. First, identifying the role of specific neural substrates in optimal performance is the first step to develop more targeted interventions. For example, if attenuated insular activation during aversive interoceptive experiences is consistent with optimal performance, one may begin to target insula modulation as a brain intervention to improve performance. Second, studies of specific neural processing pathways involved in performance in extreme environments can be used to determine which processes are important for modulating optimal performance. For example, it may be possible to use training of anticipatory processing of aversive interoceptive events as a way of improving the efficiency of deployment of effortful resources in extreme environments. Third, quantitative assessment of the contribution of different neural systems to performance in extreme environments could be used as indicators of training status or preparedness. These are just some of the possibilities for utilizing neuroscience approaches to gain a better understanding of what makes individuals perform differently when exposed to extreme environments. As a consequence, one can begin to employ a rational approach to develop strategies to improve performance in these environments.
