# Correction: Subjecting Elite Athletes to Inspiratory Breathing Load Reveals Behavioral and Neural Signatures of Optimal Performers in Extreme Environments

**DOI:** 10.1371/annotation/4d9bfe2b-0a77-470f-9260-85dd7268a032

**Published:** 2012-02-29

**Authors:** Martin P. Paulus, Taru Flagan, Alan N. Simmons, Kristine Gillis, Eric G. Potterat, Sante Kotturi, Nathaniel Thom, Douglas C. Johnson, Karl F. Van Orden, Paul W. Davenport, Judith L. Swain

Author Eric G. Potterat was incorrectly omitted from the author list. The correct author list is: Martin P. Paulus, Taru Flagan, Alan N. Simmons, Kristine Gillis, Eric G. Potterat, Sante Kotturi, Nathaniel Thom, Douglas C. Johnson, Karl F. Van Orden, Paul W. Davenport, Judith L. Swain.

Eric G. Potterat's affiliation is: 2 OptiBrain Consortium, San Diego, California, United States of America,and 6 Naval Special Warfare Group One.

The correct citation is: Paulus MP, Flagan T, Simmons AN, Gillis K, Potterat EG, et al. (2012) Subjecting Elite Athletes to Inspiratory Breathing Load Reveals Behavioral and Neural Signatures of Optimal Performers in Extreme Environments. PLoS ONE 7(1): e29394. doi:10.1371/journal.pone.0029394

Eric G. Potterat's author contributions are: Conceived and designed the experiments.

